# Predicting Factors for Metabolic Non-Response to a Complex Lifestyle Intervention—A Replication Analysis to a Randomized-Controlled Trial

**DOI:** 10.3390/nu14224721

**Published:** 2022-11-09

**Authors:** Stefan Kabisch, Nina M. T. Meyer, Caroline Honsek, Margrit Kemper, Christiana Gerbracht, Ayman M. Arafat, Ulrike Dambeck, Martin A. Osterhoff, Martin O. Weickert, Andreas F. H. Pfeiffer

**Affiliations:** 1 Department of Endocrinology and Metabolic Medicine, Campus Benjamin Franklin, Charité University Medicine, Hindenburgdamm 30, 12203 Berlin, Germany; 2 Deutsches Zentrum für Diabetesforschung e.V., Geschäftsstelle am Helmholtz-Zentrum München, Ingolstädter Landstraße 1, 85764 Neuherberg, Germany; 3 Department of Clinical Nutrition, German Institute of Human Nutrition Potsdam-Rehbruecke, Arthur-Scheunert-Allee 114-116, 14558 Nuthetal, Germany; 4 Warwickshire Institute for the Study of Diabetes, Endocrinology and Metabolism, The ARDEN NET Centre, ENETS CoE, University Hospitals Coventry and Warwickshire NHS Trust, Coventry CV2 2DX, UK; 5 Centre of Applied Biological & Exercise Sciences (ABES), Faculty of Health & Life Sciences, Coventry University, Coventry CV1 5FB, UK; 6 Translational & Experimental Medicine, Division of Biomedical Sciences, Warwick Medical School, University of Warwick, Coventry CV4 7AL, UK

**Keywords:** diabetes mellitus type 2, prediabetes, diabetes prevention, impaired fasting glucose, impaired glucose tolerance, insoluble dietary fiber, insulin sensitivity, insulin secretion, NAFLD, response prediction

## Abstract

Background: T2DM heterogeneity affects responsiveness to lifestyle treatment. Beta-cell failure and nonalcoholic fatty liver disease (NAFLD) independently predict T2DM, but NAFLD inconsistently predicts metabolic response to lifestyle intervention. Aim: We attempt to replicate a prediction model deducted from the Tübinger Lifestyle Intervention Program by assessing similar metabolic factors to predict conversion to normal glucose regulation (NGR) in a comparable lifestyle intervention trial. Methods: In the Optimal Fiber Trial (OptiFiT), 131 Caucasian participants with prediabetes completed a one-year lifestyle intervention program and received a fiber or placebo supplement. We compared baseline parameters for responders and non-responders, assessed correlations of major metabolic changes and conducted a logistic regression analysis for predictors of remission to NGR. Results: NGR was achieved by 33 participants, respectively. At baseline, for the placebo group only, 1 h and 2 h glucose levels, glucose AUC and Cederholm index predicted conversion to NGR. HOMA-beta, HOMA-IR or liver fat indices did not differ between responders and non-responders of the placebo or the fiber group. Changes in waist circumference or fatty liver index correlated with changes in glycemia and insulin resistance, but not with changes in insulin secretion. Insulin-resistant NAFLD did not predict non-response. Differences in compliance did not explain the results. Conclusions: Higher post-challenge glucose levels strongly predicted the metabolic non-response to complex lifestyle intervention in our cohort. Depending on the specific intervention and the investigated cohort, fasting glucose levels and insulin sensitivity might contribute to the risk pattern. Beta-cell function did not improve in accordance with other metabolic improvements, qualifying as a potential risk factor for non-response. We could not replicate previous data suggesting that an insulin-resistant fatty liver is a specific risk factor for treatment failure. Replication studies are required.

## 1. Introduction

The type 2 diabetes mellitus (T2DM) epidemic is continuously growing, threatening patients with premature death and morbidity, characterized by cardiovascular disease, cancer and various other long-term complications. About 50% of the disorder is attributed to genetic influences and family history, and the other half is believed to be responsive to prevention and treatment including lifestyle measures [1,2,3]. T2DM is partially caused and thereby partially treatable by changes in eating behavior, physical activity and other lifestyle factors. Large prevention trials in Caucasian and Asian cohorts have consistently demonstrated that the implementation of a complex lifestyle intervention can result in a risk reduction of diabetes incidence of about 40–60% [4,5,6,7]. These interventions include weight loss for overweight and obese prediabetes patients [8], increased physical activity and improvement of specific nutritional factors such as the reduction of saturated fat, sugars and other simple carbohydrates, alcohol and increased intake of cereal fiber [9]. In Germany, the one-year PREDIAS program combines all of these factors [10].

Recently, several publications on prediabetes and overt T2DM characterized a scheme of metabolic heterogeneity in large non-interventional cohorts by defining diabetes subphenotypes, defined by glycemic state, insulin sensitivity and insulin secretion, liver fat and BMI. Hereby, subjects with a predominant lack of insulin production and others with considerable insulin resistance are burdened with the greatest risk for diabetes complications [11,12]. Similarly, in smaller prediabetes cohorts, isolated impaired fasting glucose (IFG) leads to a lower risk of diabetes onset compared to impaired glucose tolerance (IGT), even though IFG is more strongly associated with unhealthy lifestyle, obesity and NAFLD [13,14,15,16,17]. NAFLD itself predicts prediabetes and T2DM onset in observational settings [18,19,20,21,22]. Beta-cell dysfunction is also considered an independent risk factor for progression to overt T2DM as shown by postprandial glucose levels or other measures of insulin secretion [23].

For T2DM, intervention studies have only sparsely reported results on subgroup specific responsiveness to lifestyle treatments. In previous diabetes prevention trials, stratification by IFG and IGT status indicated subtype-specific responses to lifestyle treatments, consistently showing the NAFLD-prone IFG patients achieve better improvements in response to low-fat or fiber-rich diets [24,25,26,27,28,29]. In support of these findings, elevated transaminase levels predicted diabetes progression in the control group of the J-DOIT1 trial, but a better response to lifestyle intervention [30]. Even though all of these studies indicated a particularly good response of patients with NAFLD surrogates to lifestyle treatment, none of them actually measured liver fat content [24,25,26,27,28,29].

The Tübinger Lifestyle Intervention Program (TULIP), investigating a 9-month complex lifestyle intervention (diet and exercise) in patients at elevated risk for T2DM, was the first prediabetes study directly assessing liver fat content by magnetic resonance spectroscopy. The authors confirmed impaired insulin secretion as a risk factor for metabolic inertia towards lifestyle changes, but also identified the presence of an insulin-resistant NAFLD as a high-risk condition, which contradicts the findings of earlier studies [24,25,26,27,28,29]. In TULIP, insulin sensitivity and liver fat improved in parallel to weight loss, while insulin secretion did not. In a logistic regression analysis, both low insulin secretion and insulin-resistant NAFLD qualified as independent risk factors for metabolic non-response. Based on their sophisticated risk stratification, subjects with at least one of the two risk factors have a 4-fold risk of not achieving normal glucose regulation (NGR) [31]. Up to now, this surprising result, which contradicts larger, earlier prevention studies [24,25,26,27,28,29], has not been replicated. Doing this is a difficult task, as other prediabetes studies differed in the length of intervention, characteristics of the diet, absence of exercise recommendations [4,5,6,7] and lack of MR liver fat measurements.

The Optimal Fiber Trial is a German diabetes prevention study of similar cohort size, which used a comparable intervention schema [32]. In our present paper, we aim to assess whether data from OptiFiT can replicate the findings of TULIP, using the exact same statistical approach and scheme of presentation as the original paper [31]. We expect weaker correlations between weight loss and the change in insulin secretion (compared to insulin sensitivity), as well as an independent association of low insulin secretion and insulin-resistant NAFLD with the non-remission of prediabetes.

## 2. Research Design and Methods

Recruitment, inclusion and exclusion criteria as well as the overall study design of OptiFiT have been published elsewhere [32]. OptiFiT entailed 180 adult subjects with impaired glucose tolerance (IGT), 78% of which fulfilled the definition of Metabolic Syndrome [33]. Patients with severe comorbidities or secondary origin of glycemic alterations were excluded. The study was approved by the ethics committees of the University of Potsdam and the Charité University Hospital Berlin (both under approval code EA4/192/09). OptiFiT was conducted in accordance with the Declaration of Helsinki and GCP-ICH. All subjects provided written informed consent prior to their participation. OptiFiT, the Optimal Fiber Trial, was registered at clinicaltrials.gov: NCT01681173 (first registered on 7 September 2012).

**Figure 1:**
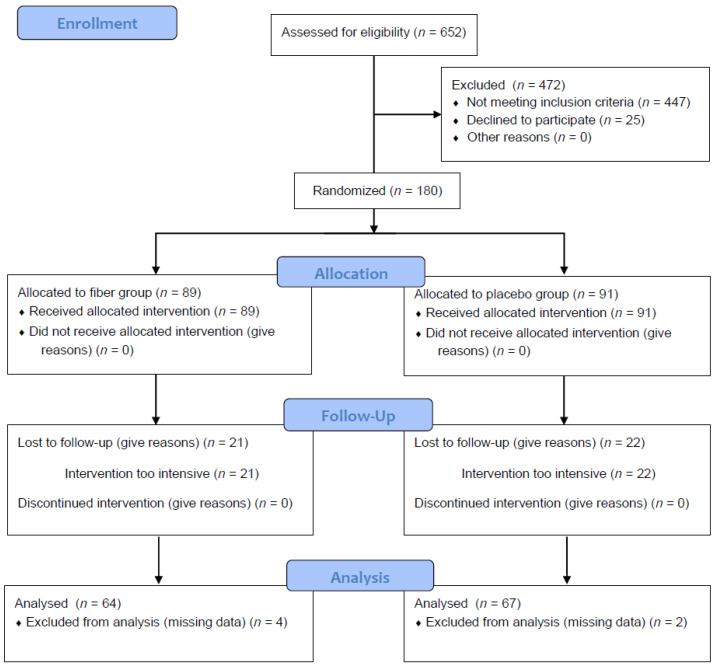
CONSORT flow diagram.

In total, 131 subjects (fiber: *n* = 64; placebo: *n* = 67; 60 ± 9 years, BMI 32.7 ± 5.9 kg/m^2^, 62% female) completed the first year of intervention and provided all data necessary for this particular post-hoc analysis for the replication of TULIP with a comparable cohort size. The selected 1-year time frame also provided the best comparability to the 9-month intervention of TULIP (Figure 1).

All participants had started our 24-month study with a modified version of the one-year lifestyle program PREDIAS, which was a structured “Treatment and Education Program for Prevention of type 2 diabetes”. PREDIAS contained group-based consultations at regular intervals and consisted of 12 individual 2 h lessons divided into a core intervention (8 lessons in 8 weeks) and booster sessions (4 lessons throughout the following 10 months) [10,34]. For OptiFiT, an adapted approach was applied by defining goals for change in diet quality in accordance with the recommendations of the German Society for Nutrition (DGE): fat intake below 30 kcal%, intake of saturated fat below 10 kcal% and intake of dietary fiber of at least 15 g/1000 kcal. We also aimed for higher levels of physical activity (240 min/week), which is above the original PREDIAS recommendation (150 min/week) [10]. We motivated our patients to consume whole-grain products, legumes, vegetables, fruits, and in particular berries, low-fat milk and meat products, soft margarines and vegetable oils rich in unsaturated fatty acids. Physical activity was monitored by pedometers and the IPAQ-2 questionnaire [35].

Our participants completed food records for four consecutive days, including one weekend day, on baseline and every six months from that. Nutrient intake was determined using the nutrition software PRODI^®^ 5.8 based on Bundeslebensmittelschlüssel 3.0 [36].

For 24 months, subjects received a blinded supplement, which contained either 70 wt% cellulose, 25 wt% hemicellulose and 3–5 wt% lignin from oat hulls (verum; Vitacel OF 560–30; Rettenmaier & Soehne Inc., Holzmuehle, Germany), adding 15 g of mainly insoluble fiber per day to the normal nutrition, or consisted of waxy maize starch with negligible content of insoluble fiber (1.6 g per day; placebo). Further details on the supplementation procedure, measurements and laboratory parameters have been given elsewhere [32].

Metabolic state was assessed by oral glucose tolerance tests (oGTT) with half-hourly capillary glucose measurements for two hours. From these tests, serum levels for insulin and c-peptide were derived every hour, and areas under the curve (AUC) for capillary glucose, insulin and c-peptide were calculated by the trapezoidal method.

For TULIP, which we wanted to replicate, subjects were classified on the basis of arbitrary cut-off values (medians) for whole-body insulin sensitivity (Matsuda index) and for insulin secretion capacity (disposition index) as well as by the validated cut-off for NAFLD (MR spectroscopy, >5.56%) [31,37,38,39]. In our study, due to budget reasons, oGTT measurements for insulin included only limited (hourly) time points; therefore, neither the disposition index nor Matsuda index could be calculated. Additionally, MR spectroscopy was only available for a small subgroup (26% of the cohort) [40]. Therefore, we used the Cederholm index [41] to quantify insulin sensitivity and the HOMA-beta to assess insulin secretion [42], as those could be calculated with our limited set of serum blood samples (0′, 60′, 120′). The fatty liver index (FLI) was calculated according to the primary publication [43]. Following the TULIP approach, we classified patients by median splits for insulin resistance and insulin secretion. The established cut-off for fatty liver (in our case: FLI > 60) defined the presence of NAFLD.

In the TULIP study, subjects were classified as responders when achieving “NGR”. In our study, subjects were also classified as “NGR” (responders) if they achieved normalization of both fasting (<90 mg/dL capillary glucose levels) and the post-challenge glucose (<140 mg/dL capillary glucose levels) after one year. All subjects who failed to achieve this normalization were labelled “prediabetes/T2DM” (non-responders). As the major inclusion criterion of our study was the presence of IGT, we tested an alternative response classification, comparing those who achieved normalization of post-challenge glucose after one year, irrespective of (non-diabetic) fasting glucose levels (“NGT”; responders), with subjects failing to achieve normalization of post-challenge glucose levels (“IGT/T2DM”, non-responders).

### Statistical Analyses

We used the Kolmogorov–Smirnov test in order to determine the normal distribution of our data. Given the frequent absence of normal distribution, we decided to conduct non-parametric tests (Mann–Whitney tests) to compare major metabolic parameters at baseline and indicators of lifestyle compliance between the responders and non-responders for both intervention arms separately. For correlations, the non-parametric Spearman approach was chosen. Comparisons between post-hoc defined risk groups were made by ANCOVA (adjusted for sex, age, baseline levels), following the original publication of TULIP [31]. In addition, in analogy to the TULIP analysis, a multivariate logistic regression model was used to identify binary factors predicting non-response [31]. We included age, BMI, waist circumference, fasting and 2 h glucose, liver fat (fatty liver index), insulin secretion (HOMA-beta) and insulin sensitivity (Cederholm index) as continuous variables, and sex and the presence of insulin-resistant fatty liver (Cederholm index below the median and FLI > 60) as independent categorial variables.

All data were presented as means ± standard deviation. The results were considered significantly different if *p* < 0.05. All statistical analyses were performed using SPSS for Windows program version 25.0 (SPSS Inc., Chicago, IL, USA).

## 3. Results

### 3.1. Cohort Structure

**Figure 2:**
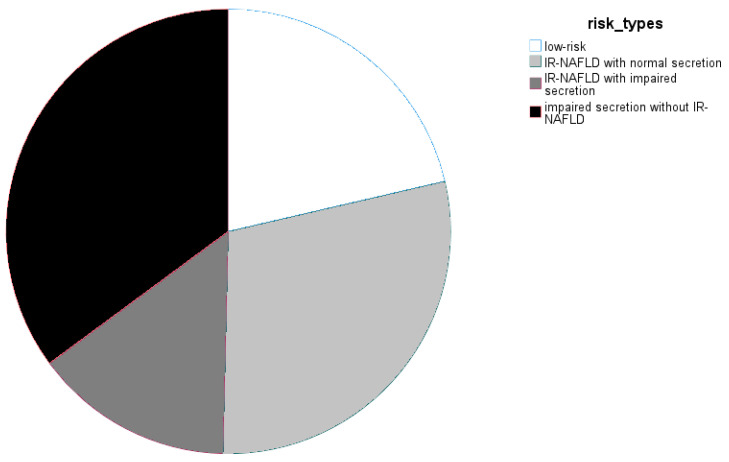
Cohort structure of OptiFiT according to the stratification schema, derived from the TULIP study.

At baseline, out of the 131 eligible subjects, 65 subjects were classified as insulin-deficient (HOMA-beta < 74.6) and 66 as insulin-sufficient (HOMA-beta > 74.6); 65 participants were considered to be whole-body insulin-resistant (Cederholm < 35.1) and 66 as whole-body insulin-sensitive (Cederholm > 35.1). NAFLD was assumed in 93 subjects (FLI > 60), while 38 subjects had FLI values that were either indicative of the absence of NAFLD (<30) or inconclusive for classification (30–60). In total, 28 subjects (21%) had neither insulin-resistant NAFLD nor an insulin secretion deficit and would resemble the “low-risk” phenotype of TULIP, while the remaining 103 subjects combined (79%) were representative of the TULIP “high-risk” phenotype. In total, 57 subjects with NAFLD were insulin-resistant. Of those, 19 had an additional beta-cell dysfunction (HOMA-beta < 74.6), while 38 had an insulin-resistant NAFLD with preserved insulin secretion capacity [31]. See Figure 2 for an overview of the cohort structure.

### 3.2. Intervention Effects and Factors Predicting Achievement of NGR

After one year, in the fiber group, NGR was achieved by 14 subjects. In the placebo group, one-year conversion to NGR was found in 19 subjects. Although there was no significant difference in the frequency of NGR between the fiber and placebo groups, there were significant differences in HbA1c and 2 h glucose levels as shown before [32].

Lower 1 h and 2 h glucose levels and glucose AUC as well as a higher Cederholm index were predictive for conversion to NGR (Table 1, first columns) in the placebo group. In the fiber group, lower 2 h glucose levels, exclusively and only trend-wise, were predictive for conversion to NGR (Table 1, middle columns). When combining the fiber and placebo groups, lower 2 h glucose and glucose AUC were significantly, and a higher Cederholm index was trend-wise associated with regression to NGR (Table 1, last columns). The results for both groups (separately and combined) were similar, when evaluating the conversion to NGT rather than NGR (Table A1). Neither in the two intervention arms separately, nor in the total cohort, did measures for fasting insulin sensitivity (HOMA-IR), insulin secretion (HOMA-beta) and liver fat (fatty liver index, estimated liver fat) differ significantly between responders and non-responders.

Interventional changes of total energy, fat and fiber intake, body weight and physical activity were not significantly different between metabolic outcome groups (Table 2).

Interventional changes in waist circumference (as a surrogate for visceral fat) correlated with changes of the Cederholm index in the fiber group, and with both changes of the Cederholm index and fasting glucose in the total cohort. Changes in FLI correlated with changes of the Cederholm index in both groups and the full cohort as well as with 2 h glucose in the full cohort. Insulin secretion capacity (HOMA-beta) was the only metabolic variable not showing any linkage to changes in body fat depots in any study group nor the full cohort (Table 3A–C).

In the multivariate logistic regression analysis, neither fasting glucose, liver fat content, insulin resistance nor insulin secretion were predictive markers for metabolic response, but only 2 h glucose levels significantly predicted the response to lifestyle intervention within the total cohort (Table 4).

Finally, we assessed if a classification of risk phenotypes according to insulin secretion and/or insulin-resistant fatty liver disease indicated different changes in 2 h glucose levels, when comparing the phenotypes. First, we classified all subjects in analogy to the TULIP study: subjects with insulin secretion capacity (HOMA-beta) below median and/or insulin-resistant NAFLD (insulin sensitivity (Cederholm index) below median and FLI > 60) were considered as high risk, and all others as low risk (model 1). As insulin resistance and fatty liver index (but not insulin secretion) correlated with changes in body fat and glycemia, we hypothesized that an insulin-resistant NAFLD might not be an independent risk factor. Therefore, we tested an additional classification where only insulin secretion failure—strongly reflected by 2 h glucose—was defined as a high-risk phenotype and all subjects with normal insulin secretion—including those with insulin-resistant NAFLD—were considered as low risk (model 2). ANCOVAs on the relative change of 2 h glucose levels (all models including sex, age and treatment group) did not show a significant difference between respective high- and low-risk groups (adjusted model 1: *p* = 0.512, adjusted model 2: *p* = 0.358), nor an interaction with sex and the treatment group (interaction model 1: *p* = 0.142; interaction model 2: *p* = 0.146).

## 4. Discussion

Our analysis is the first attempt to replicate the risk categorization of TULIP. Perhaps surprisingly, in our study, NAFLD—either with or without concomitant insulin resistance—did not qualify as a high-risk phenotype. Equally opposite to the results from TULIP, higher fasting glucose levels turned out to be neutral or even beneficial concerning metabolic success in OptiFiT [24,25,26,27,28,29,44]. Nevertheless, we confirm a major finding of previous cohort and intervention studies: impaired insulin secretion capacity determines non-response to lifestyle intervention. This is reflected by the absence of correlations between the change in insulin secretion (HOMA-beta) with interventional changes in body fat depots. In support of this, higher levels of 2 h glucose—predominantly mirroring peripheral insulin resistance and/or failure in 2nd-phase insulin secretion—are a strong predictor for failure to achieve NGR in our trial [45,46].

According to previous large-scale RCTs on diabetes prevention with thousands of patients from Finland, the USA, China, Japan and India, respectively, lifestyle intervention is capable of reducing diabetes risk by up to 50% in the long-term perspective, to improve quality of life and to prevent late complications [4,5,6,7]. These studies further highlighted that the *metabolic* heterogeneity of patients results in *clinical* heterogeneity in interventional outcomes: not all subgroups of subjects achieve glycemic normalization, despite sufficient compliance to treatment. While the existence of different risk types is a fact, the distinct definitions of those phenotypes are still disputed. The above-mentioned large intervention trials consistently showed that the IFG-IGT phenotype (bearing a higher risk for NAFLD than NFG-IGT) is especially responsive to a low-fat diet and increased physical activity [24,25,26,27,28,29].

IFG, either as an isolated feature or combined with glucose intolerance, is strongly connected to a weakened 1st-phase insulin secretion capacity [47,48]. Isolated glucose intolerance is identified as a risk factor for non-response [24,25,26,27,28,29], as 2 h glucose levels are reported to be major drivers of treatment failure [5]. IGT—be it isolated or combined with IFG—mirrors declining whole-body insulin sensitivity and reduced 2nd-phase insulin secretion, and bears a higher risk for progression, especially due to its tendency towards metabolic non-response [14,15,16,17].

The TULIP study pointed out that low insulin secretion capacity (defined by the 1st-phase insulin secretion marker “disposition index”) and insulin-resistant fatty liver are independent risk factors for lifestyle non-response [31]. Both beta-cell failure and (insulin-resistant) fatty liver have been considered as risk factors for disease progression in prediabetes cohorts *without* treatment; NAFLD was seen as a problematic state especially when combined with IFG [46,47,49]. *Intervention* studies soon confirmed and emphasized that beta-cell dysfunction and insulin resistance are indeed major players in counteracting the induction of metabolic improvements [50,51]. In our current analysis, insulin secretion was not connected to any glycemic or anthropometric response to lifestyle treatment, supporting the notion of a response-determining risk factor, despite a lack of significance in the logistic regression analysis. IGT cohorts such as OptiFiT predominantly include insulin-deficient subjects, possibly masking the strong disadvantage of this metabolic feature in comparison to underrepresented subjects with preserved secretion capacity. However, it seems logical that patients with early beta-cell dysfunction as a potentially genetic feature (as it is already present in prediabetes) cannot improve the metabolism despite losing weight and improving diet quality.

Neither insulin resistance, NAFLD nor their combination predicted non-response in our analysis. Reductions in waist circumference and FLI were correlated with improvements in the Cederholm index. Despite being risk factors for T2DM onset under *observational* conditions, it seems that these factors can be at least partially ameliorated by lifestyle measures, even in a high-risk cohort such as OptiFiT.

A possible explanation for the inconsistencies between OptiFiT and TULIP may be found in their cohort structure. OptiFiT exclusively recruited subjects with IGT (isolated and combined with IFG), while TULIP also contained subjects with isolated IFG. The latter usually makes up about 30–40% of all prediabetes cases. Usually, patients with isolated IFG show higher levels of liver fat than subjects with isolated IGT [13]. Surprisingly, this was not the case in the TULIP cohort [52], while OptiFiT replicated this difference between IFG-IGT and isolated IGT [28,44].

In both TULIP and OptiFiT, the insulin secretion deficit was defined by the median of the respective secretion index (disposition index, HOMA-beta), i.e., in both studies, 50% of the cohort were considered insulin-deficient. In TULIP, only 10% (12/120) had an insulin-resistant NAFLD with normal insulin secretion [31], while in OptiFiT, 29% (38/131) showed this phenotype. Thus, the high-risk phenotype in TULIP—rather than in OptiFiT—is strongly dominated by patients with secretion deficit (with or without additional insulin-resistant NAFLD). Earlier reports on the regional distribution of prediabetes subtypes within Germany support the plausibility of these differences [53].

By having a sufficiently large proportion of patients with insulin-resistant NAFLD, OptiFiT specifically might be able to demonstrate that this phenotype is not resistant to lifestyle intervention. This does not contradict cohort studies showing the independent contribution of NAFLD to diabetes risk, as they assess “natural progression” rather than *treatment* responsiveness [54,55].

Even though we cannot replicate the TULIP finding on the high-risk potential of an insulin-resistant NAFLD, our current analysis of OptiFiT supports the findings of DPP and DREAM, highlighting that the response to interventions mainly depends on the glycemic state itself. Higher levels of 2 h glucose predict treatment failure, while isolated or the additional impairment of fasting glucose increases the chances for treatment success [23,24,25,26,27]. Similarly, IFG-IGT patients in OptiFiT were characterized by higher post-challenge glucose levels than NFG-IGT subjects, but they had a better outcome. The fiber supplementation seemed to compensate for the additional metabolic disadvantage, resulting in a significant interaction effect (IFG x treatment) [28,44].

Previous prediabetes studies had proposed to use 1 h glucose levels as an additional or even superior risk parameter for diabetes progression [56]. Our data imply that under interventional rather than observational conditions, this diagnostic superiority is absent.

In both TULIP and OptiFiT, all subjects were motivated to increase their physical activity, to reduce fat intake and to increase their fiber intake from daily food sources. Apart from fiber intake, any other lifestyle aspect might have affected the metabolic outcome: weight loss, reduction in fat intake and increased physical activity [57]. Responders and non-responders did not differ in these intervention-induced changes, ruling out further explanatory factors for non-response. Compliance was not a relevant effect modulator. However, poor compliance to fiber-rich diets has been consistently reported from large diabetes prevention trials [58] and in OptiFiT [32], underlining our focus on insoluble cereal fiber in all efforts (natural food sources, fortification and supplementation) to reduce diabetes incidence [59,60]. The limited compliance to the recommendation of fiber-rich food might explain the impact of supplementation: in the placebo group, NGR and non-NGR subjects differed in various glycemic baseline parameters. In the fiber group, 2 h glucose at baseline was the only (trend-wise) discriminant between subjects achieving NGR or non-NGR. On the other hand, in the fiber group, improvements in NAFLD and visceral fat amount were more strongly linked to improved insulin sensitivity compared to placebo. This mirrors the accentuated effectiveness of the fiber treatment in IFG-IGT and/or obese subjects as previously shown [28,44].

There are, however, some limitations to our analysis. Compared to other large lifestyle RCTs, OptiFiT was a rather small trial, limiting power for comparisons. Furthermore, male subjects were underrepresented. Nevertheless, our cohort was of a similar size as the prediabetes subgroup of TULIP and the intervention entailed a 30% low-fat diet and exercise recommendation of similar duration. These properties provided sufficient comparability [31].

The blood sampling protocol for OptiFiT was very restrictive due to budget reasons. By forfeiting 30′ sampling for insulin levels, indices for post-challenge insulin secretion (e.g., disposition index) and several insulin sensitivity indices (such as Matsuda) could not be assessed. We therefore used the Cederholm index to assess insulin sensitivity, which provided predictive properties comparable to the Matsuda [61,62,63]. HOMA-beta as an alternative to the disposition index is commonly used as a cross-sectional and prospective surrogate of insulin secretion [11,64,65,66].

Additionally, liver fat measurements were only available in a small subgroup which required the use of the FLI to describe the NAFLD status at baseline. For this purpose alone, the FLI is sufficiently reliable and commonly used as a diabetes predictor in epidemiology [18,19,20,21,22,44]. The FLI provides a similar predictive power compared to sonography [67,68]. Changes in liver fat due to a low-fat diet can be described on the basis of changes in FLI [69].

We experienced a one-year drop-out rate of 24%. This is comparable to the 9-month TULIP and should not explain differences in the results between TULIP and OptiFiT [31]. In OptiFiT, the onset of T2DM was a drop-out criterion, but only one case did not take part in the one-year visit. Drop-outs similarly limit the prediction quality of both the TULIP and OptiFiT models.

By using 4-day food records, we were able to evaluate dietary compliance and could outrule a considerable misinterpretation by under-/overreporting or under-/overeating in the absence of valid biomarkers for dietary protocol adherence. The parallel use of pedometers and EPAQ-2 questionnaires assured the analysis of physical activity based on both objective and subjective parameters. Compliance to recommendations for both diet and physical activity was moderate, but comparable between intervention groups, responders and non-responders, as well as TULIP and OptiFiT. The well-accepted OptiFiT fiber supplement provided the unique chance to significantly raise fiber intake.

In the same way as the TULIP analysis, our assessment included sex as a potential effect modulator, but was not powered for a sex-stratified approach. The prevalence of NAFLD and prediabetes subtypes differed between sexes, but as the statistical procedure clearly split up these phenotypes, the modulating effect of sex was minimized [70].

## 5. Conclusions

In summary, insulin resistance, measures of liver fat content and the insulin-resistant NAFLD did not appear as factors modulating the risk for non-response and might be considered as both therapeutic targets and predictors for successful lifestyle treatment.

We further replicated previous findings on glycemic predictors for the metabolic non-response to lifestyle treatment. Fasting glucose and—more consistently for different treatments—post-challenge 2 h glucose determined the chance to achieve NGR or NGT. Insulin secretion capacity did not change in parallel to measures of excess fat depots. Thus, impaired insulin secretion seems to consistently represent a major obstacle with respect to lifestyle-dependent metabolic improvements.

We warrant detailed subgroup analyses for other non-pharmaceutical prevention trials will clearly determine the risk factors for T2DM progression despite sufficient compliance.

## Figures and Tables

**Table 1:** Characteristics of responders and non-responders (NGR) at study entry; Mann–Whitney tests for comparison of NGR and non-NGR subgroups.

Characteristics of Participants at Study Entry	Placebo Arm			Fiber Arm			Full Cohort		
	NGR (*n* = 19)	No NGR (*n* = 48)	*p*-Value	NGR (*n* = 14)	No NGR (*n* = 50)	*p*-Value	NGR (*n* = 33)	No NGR (*n* = 98)	*p*-Value
**Cohort Structure**									
Age	61 ± 10	60 ± 9	n.s.	58 ± 10	60 ± 9	n.s.	59 ± 10	60 ± 9	n.s.
Sex	63%	48%	n.s.	64%	74%	n.s.	64%	61%	n.s.
**Anthropometry**									
Weight (kg)	91.8 ± 22.3	94.5 ± 20.7	n.s.	90.0 ± 12.5	87.3 ± 16.6	n.s.	91.0 ± 18.5	90.8 ± 19.0	n.s.
BMI (kg/m^2^)	34.3 ± 8.1	33.0 ± 6.0	n.s.	32.0 ± 4.9	32.0 ± 5.2	n.s.	33.3 ± 6.9	32.5 ± 5.6	n.s.
Waist circumference (cm)	106.6 ± 18.1	106.6 ± 13.4	n.s.	105.3 ± 12.0	101.8 ± 13.0	n.s.	106.1 ± 15.6	104.2 ± 13.3	n.s.
Hip circumference (cm)	115.5 ± 15.9	114.0 ± 13.0	n.s.	111.7 ± 10.6	111.5 ± 12.9	n.s.	113.8 ± 13.9	112.7 ± 12.9	n.s.
WHR	0.92 ± 0.09	0.94 ± 0.08	n.s.	0.94 ± 0.07	0.92 ± 0.08	n.s.	0.93 ± 0.08	0.93 ± 0.08	n.s.
BIA—Body fat (%)	37.2 ± 10.3	35.3 ± 7.7	n.s.	32.6 ± 9.4	37.7 ± 7.7	n.s.	35.7 ± 1.01	36.6 ± 7.7	n.s.
**Glycemia**									
Fasting glucose (mg/dL)	87.6 ± 9.0	92.7 ± 10.3	n.s.	91.6 ± 10.4	89.8 ± 10.7	n.s.	89.3 ± 9.7	91.2 ± 10.5	n.s.
1-h glucose (mg/dL)	183.5 ± 32.3	206.6 ± 29.4	0.011	207.0 ± 29.8	197.5 ± 34.1	n.s.	193.5 ± 33.0	202.0 ± 32.0	n.s.
2-h glucose (mg/dL)	150.3 ± 14.2	164.8 ± 18.8	0.002	151.9 ± 12.1	159.4 ± 16.8	n.s. (*p* = 0.102)	151.0 ± 13.2	162.0 ± 17.9	0.001
Glucose AUC (mg/dL*min)	19,391.6 ± 2045.4	21,271.5 ± 2236.8	0.004	20,399.0 ± 2799.3	20,635.9 ± 2584.1	n.s.	19,828.2 ± 2410.1	20,950.4 ± 2426.8	0.020
**Insulin Resistance and Beta-Cell Function**									
Fasting insulin (mU/L)	9.0 ± 3.8	10.4 ± 6.3	n.s.	7.8 ± 3.6	9.2 ± 4.6	n.s.	8.5 ± 3.7	9.8 ± 5.5	n.s.
Fasting c-Peptide (µg/L)	1.7 ± 0.5	1.7 ± 0.8	n.s.	1.6 ± 0.8	1.6 ± 0.7	n.s.	1.6 ± 0.6	1.7 ± 0.7	n.s.
Cederholm	41.1 ± 10.3	35.4 ± 12.9	0.032	39.8 ± 15.1	36.7 ± 10.6	n.s.	40.5 ± 12.4	36.0 ± 11.7	n.s. (*p* = 0.052)
HOMA-beta	85.7 ± 39.6	80.9 ± 40.4	n.s.	62.0 ± 29.7	79.3 ± 35.7	n.s.	75.7 ± 37.2	8.1 ± 37.9	n.s.
**NAFLD**									
Fatty liver index	70 ± 32	72 ± 25	n.s.	71 ± 26	67 ± 26	n.s.	71 ± 29	70 ± 25	n.s.

**Table 2:** Changes in lifestyle parameters during the one-year PREDIAS intervention; Mann–Whitney tests for comparison of NGR and non-NGR subgroups.

Lifestyle Changes over One Year	Full Cohort		
	NGR (*n* = 33)	No NGR (*n* = 98)	*p*-Value
**Eating Behavior**			
Change in body weight (kg)	−3.6 ± 5.1	−3.3 ± 5.7	n.s.
Change in energy intake (kcal)	−275 ± 547	−273 ± 474	n.s.
Change in fat intake (%)	−4.1 ± 7.4	−1.8 ± 6.4	n.s.
Change in dietary fiber intake (g)	0 ± 8	0 ± 8	n.s.
Change in supplemented fiber intake (g)	6 ± 9	7 ± 10	n.s.
**Physical Activity**			
Change in steps per day (n)	−278 ± 2692	629 ± 2865	n.s.

**Table 3:** Correlation of fold changes in body fat compartments and metabolic outcomes; (**A**): placebo group, (**B**): fiber group; (**C**): total cohort; Spearman correlations. Significant results are printed in bold; *: *p* < 0.05; **: *p* < 0.01; ***: *p* < 0.001.

(A)						
Variable	Change in Body fat_BIA_		Change in Waist Circumference		Change in FLI	
	ϱ	*p*	ϱ	*p*	ϱ	*p*
Change in fasting glucose	−0.081	0.559	0.224	0.068	0.104	0.405
Change in 2 h glucose	0.034	0.807	0.075	0.545	0.217	0.080
Change in Cederholm index	−0.171	0.221	−0.149	0.232	**−0.320**	0.009 **
Change in HOMA-beta	0.077	0.586	0.028	0.824	0.141	0.258
(**B**)						
**Variable**	**Change in Body fat_BIA_**		**Change in Waist Circumference**		**Change in FLI**	
	**ϱ**	***p***	**ϱ**	***p***	**ϱ**	***p***
Change in fasting glucose	0.159	0.265	0.223	0.077	0.089	0.487
Change in 2 h glucose	0.030	0.836	0.049	0.699	0.176	0.164
Change in Cederholm index	−0.150	0.293	**−0.300**	0.017 *	**−0.489**	<0.001 ***
Change in HOMA-beta	0.155	0.276	−0.185	0.143	−0.049	0.699
(**C**)						
**Variable**	**Change in Body fat_BIA_**		**Change in Waist Circumference**		**Change in FLI**	
	**ϱ**	***p***	**ϱ**	***p***	**ϱ**	***p***
Change in fasting glucose	0.038	0.700	**0.233**	0.007 **	0.104	0.241
Change in 2 h glucose	0.020	0.837	0.052	0.555	**0.201**	0.022 *
Change in Cederholm index	−0.144	0.145	**−0.216**	0.014 *	**−0.403**	<0.001 ***
Change in HOMA-beta	0.098	0.320	−0.065	0.465	0.057	0.523

**Table 4:** Likelihood ratios for multivariate nominal logistic regression model for prediction of conversion to normal glucose regulation. Sex and Cederholm x FLI were analyzed as categorical variables. Significant results are printed in bold; *: *p* < 0.05.

	Placebo Group		Fiber Group		Total Cohort	
Variable	Likelihood Ratio *χ*^2^	*p* Value	Likelihood Ratio *χ*^2^	*p* Value	Likelihood Ratio *χ*^2^	*p* Value
Sex	0.062	0.803	0.100	0.751	0.133	0.715
Age	0.204	0.652	0.474	0.491	0.000	0.984
BMI	0.510	0.475	2.628	0.105	0.020	0.886
Waist circumference	0.014	0.907	2.545	0.111	0.327	0.567
Fasting glucose levels	0.782	0.377	0.233	0.629	0.033	0.856
2 h glucose levels	3.360	0.067	2.282	0.131	**5.588**	0.018 *
HOMA-beta	0.402	0.526	2.842	0.092	0.040	0.842
Cederholm index	0.089	0.765	0.474	0.491	0.073	0.787
Fatty liver index (FLI)	0.101	0.751	0.314	0.575	0.001	0.971
Cederholm × FLI	0.794	0.373	0.371	0.543	0.103	0.748

## Data Availability

Data sets are available by request to the corresponding author.

## Abbreviations

AUC	area under the curve
BIA	bioelectric impedance analysis
FLI	fatty liver index
HIC	hepatic insulin clearance
HOMA_IR_	homeostasis model assessment insulin resistance index
HOMA-beta	homeostasis model assessment insulin for beta cell function
IFG	impaired fasting glucose
IGT	impaired glucose tolerance
ISI_ffa_	insulin sensitivity index of blood free fatty acids
NAFLD	nonalcoholic fatty liver disease
NFG	normal fasting glucose
NGR	normal glucose regulation
NGT	normal glucose tolerance
OptiFiT	Optimal Fiber Trial for diabetes prevention
OGTT	oral glucose tolerance test
T2DM	type 2 diabetes mellitus

## Appendix A

**Table A1:** Characteristics of responders and non-responders (NGT) at study entry; Mann–Whitney tests for comparison of NGT and non-NGT subgroups.

Characteristics of Participants at Study Entry	Placebo Arm			Fiber Arm			Full Cohort		
	NGT (*n* = 23)	No NGT (*n* = 44)	*p*-Value	NGT (*n* = 23)	No NGT (*n* = 41)	*p*-Value	NGT (*n* = 46)	No NGT (*n* = 85)	*p*-Value
**Cohort Structure**									
Age	61 ± 10	60 ± 9	n.s.	56 ± 10	62 ± 8	0.018	58 ± 10	61 ± 9	n.s.
Sex	57%	50%	n.s.	78%	68%	n.s.	67%	59%	n.s.
**Anthropometry**									
Weight (kg)	92.2 ± 21.7	94.5 ± 20.9	n.s.	91.1 ± 13,1	86.1 ± 16.9	n.s.	91.7 ± 17.7	90.4 ± 19.4	n.s.
BMI (kg/m^2^)	33.7 ± 7.7	33.2 ± 6.0	n.s.	33.0 ± 4.7	31.5 ± 5.3	n.s.	33.3 ± 6.3	32.3 ± 5.7	n.s.
Waist circumference (cm)	106.8 ± 18.2	106.5 ± 12.8	n.s.	105.8 ± 12.0	100.8 ± 13.0	n.s.	106.3 ± 15.2	103.8 ± 13.1	n.s.
Hip circumference (cm)	115.1 ± 15.9	114.0 ± 12.7	n.s.	114.6 ± 11.3	109.8 ± 12.8	n.s.	114.8 ± 13.6	112.0 ± 12.8	n.s.
WHR	0.93 ± 0.08	0.94 ± 0.09	n.s.	0.92 ± 0.07	0.92 ± 0.09	n.s.	0.93 ± 0.07	0.93 ± 0.09	n.s.
BIA—Body fat (%)	36.8 ± 10.2	35.4 ± 7.4	n.s.	37.5 ± 8.7	36.7 ± 8.0	n.s.	37.1 ± 9.4	36.0 ± 7.7	n.s.
**Glycemia**									
Fasting glucose (mg/dL)	87.7 ± 9.2	93.1 ± 10.2	n.s. (*p* = 0.053)	91.7 ± 9.2	89.3 ± 11.3	n.s.	89.7 ± 9.3	91.3 ± 10.8	n.s.
1 h glucose (mg/dL)	189.8 ± 34.8	205.4 ± 29.0	n.s. (*p* = 0.069)	201.7 ± 28.6	189.4 ± 35.8	n.s.	195.7 ± 32.1	202.0 ± 32.5	n.s.
2 h glucose (mg/dL)	149.4 ± 13.2	166.6 ± 18.5	<0.001	153.0 ± 12.9	160.4 ± 17.3	n.s. (*p* = 0.078)	151.2 ± 13.0	163.6 ± 18.1	<0.001
Glucose AUC (mg/dL*min)	19,777.6 ± 2261.9	21,257.9 ± 2225.2	0.018	20,288.8 ± 2430.9	20,752.8 ± 2721.0	n.s.	20,039.2 ± 2336.1	21,017.7 ± 2470.9	0.027
**Insulin Resistance and Beta-Cell Function**									
Fasting insulin (mU/L)	8.7 ± 4.0	10.7 ± 6.3	n.s.	8.7 ± 3.8	9.0 ± 4.7	n.s.	8.7 ± 3,9	9.9 ± 5.6	n.s.
Fasting c-Peptide (µg/L)	1.6 ± 0.6	1.7 ± 0.7	n.s.	1.7 ± 0.8	1.6 ± 0.7	n.s.	1.7 ± 0.7	1.6 ± 0.7	n.s.
Cederholm	40.8 ± 10.7	35.0 ± 12.9	0.027	38.6 ± 12.1	36.7 ± 11.6	n.s.	39.7 ± 11.4	35.8 ± 12.2	0.034
HOMA-beta	79.1 ± 39.9	83.9 ± 40.3	n.s.	73.1 ± 30.8	76.9 ± 37.4	n.s. (*p* = 0.093)	76.1 ± 35.4	80.5 ± 38.9	n.s.
**NAFLD**									
Fatty liver index	70 ± 32	72 ± 24	n.s.	73 ± 24	65 ± 27	n.s.	72 ± 28	69 ± 26	n.s.
